# A Novel Compound Heterozygous Mutation in Progressive Familial Intrahepatic Cholestasis (PFIC) 4: A Rare Case Report With Literature Review

**DOI:** 10.7759/cureus.73927

**Published:** 2024-11-18

**Authors:** Deepika Pandey, Sanjay Chandnani, Harsh Gandhi, Mavuri Vishal, Pravin M Rathi

**Affiliations:** 1 Department of Gastroenterology, Topiwala National Medical College & BYL Nair Charitable Hospital, Mumbai, IND

**Keywords:** ana: antinuclear antibody, anti-lkm: anti-liver kidney microsomal antibody, bric: benign recurrent intrahepatic cholestasis, hcc: hepatocellular carcinoma, nsg: next-generation sequencing, pfic: progressive familial intrahepatic cholestasis, sgot: serum glutamic oxaloacetic transaminase, sgpt: serum glutamic pyruvate transaminase, sma: smooth muscle antibody, tjp2: tight-junction protein 2

## Abstract

A 12-year-old female, resident of western India, presented with a history of pruritus associated with jaundice for two months. On presentation, she had icterus with mild palpable hepatomegaly. Investigations revealed direct hyperbilirubinemia and elevated transaminases, while gamma-glutamyl transferase levels were normal. Serology for anti-hepatitis A, E, B, and C were negative. Autoimmune markers such as antinuclear antibody, smooth muscle antibody, and anti-liver kidney microsomal antibody were negative. Serum IgG levels were within the normal range. A normal magnetic resonance cholangiopancreatography ruled out any ductal abnormalities. A liver biopsy was also conducted but proved to be inconclusive. Despite extensive workup, the diagnosis remained unclear. However, genetic testing through whole exome sequencing identified a novel compound heterozygous variation, a novel in exon 5 and exon 4 of the Tight-Junction Protein 2 gene, and confirmed the diagnosis of cholestatic liver disease as progressive familial intrahepatic cholestasis type 4. This case highlights the importance of genetic testing for diagnosing cholestatic liver diseases, especially when conventional tests do not provide a clear diagnosis. Whole exome sequencing revealed a novel mutation in the TJP2 gene, ultimately confirming the diagnosis of PFIC4.

## Introduction

Progressive familial intrahepatic cholestasis (PFIC) can become symptomatic either in infancy or in adulthood, and rapid progression to severe liver disease may be seen [1-2]. PFIC is characterized by poor bile transport and production. In young individuals, PFIC instances with genetic confirmation account for 12% of cholestatic disorders [3]. All variations of PFIC are inherited autosomal recessively and are categorized according to distinct abnormalities in bile transporters. The higher risk of hepatocellular carcinoma (HCC) associated with PFIC is related to tight junction protein (TJP2) gene mutation. TJP2 is a multifaceted protein interacting with various cell signaling molecules, the actin cytoskeleton, and components of gap, tight, and adherens junctions. It also inhibits the Wnt signaling pathway, decreases cell proliferation, and encourages apoptosis [4].

## Case presentation

A 12-year-old female, born out of a non-consanguineous marriage, presented with a history of itching all over the body for three months, high-colored urine followed by yellow discoloration of eyes, and pale-colored stools for two months. The itching was initially localized on the palms and soles, gradually involving the entire body, and disturbed sleep. She started noticing high-colored urine followed by yellowish discoloration of the eye with pale stools one month after the onset of itching. There was no history of fever, prodromal symptoms, abdominal pain, or distention. There was no history of hematemesis, GI blood loss, any other bleeding manifestation, or altered sensorium. She denied the use of complementary and alternative medication or over-the-counter medication. There was no history of similar complaints in the past or other family members with similar illnesses.

**Figure 1:**
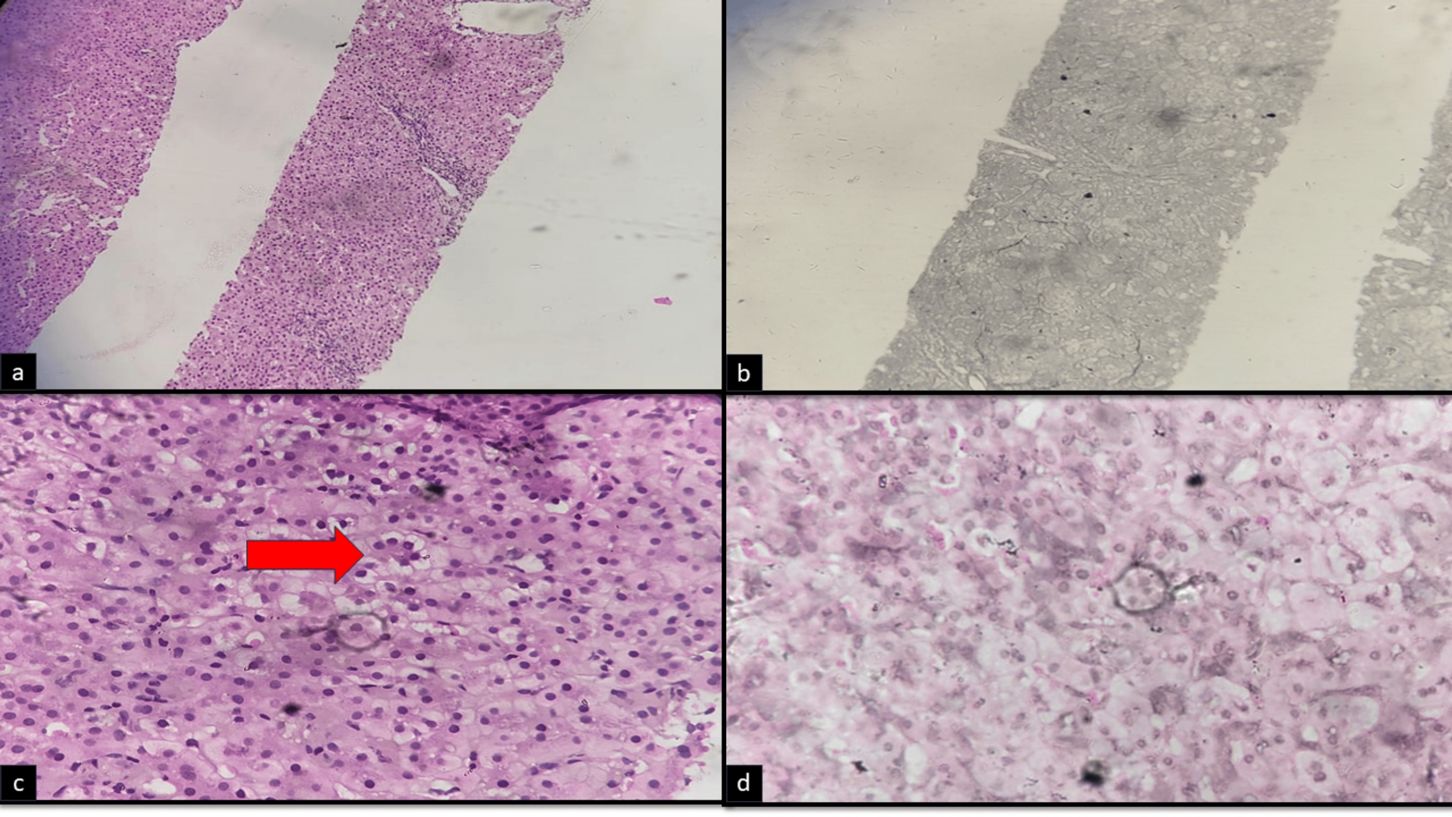
Showing histopathology of liver: a. Hematoxylin and eosin (H&E) staining of liver tissue seen in low power magnification (10x) showing normal liver architecture with mild portal widening and no evidence of bridging fibrosis with intrahepatic cholestasis.; b. Reticulin stain on liver biopsy showing no fibrosis on biopsy on low power magnification (10x); c. Hematoxylin and eosin (H&E) staining of liver tissue seen in high power magnification (40x) Bile ductule visualized in the portal area with multiple rosette formation (red arrow), no evidence of fibrosis on high power magnification (40x); d: Masson-Fontana staining on high power magnification showing perinuclear gray pigment within the cell.

On general examination, the patient was of normal weight for age (10-20 percentile), and icterus was present. On systemic examination, there was mild hepatomegaly without splenomegaly, and the liver span was 15 cm. Blood investigation revealed hemoglobin 9.6 g/dl and a total leukocyte count of 8400/microliter and platelet counts of 388 x 109/L. Biochemical investigations revealed elevated liver function tests (LFT) with conjugated hyperbilirubinemia, cholestatic pattern, preserved albumin globulin ratio, international normalized ratio (INR), and normal gamma-glutamyl transferase (GGT). Ultrasound of the abdomen with hepato-portal Doppler suggested mild hepatomegaly (13.5 cm) with an unremarkable biliary system. Serology for viral hepatitis was negative for Hepatitis A, B, C, and E. Serum anti-nuclear antibody (ANA), anti-smooth muscle antibody (ASMA), and anti-mitochondrial antibody (AMA) were negative, and serum immunoglobulin G (IgG) was in the normal range. Her Wilson’s disease workup with slit-lamp examination for the Kayser-Fleisher ring was negative, and her serum ceruloplasmin levels were normal. A liver biopsy showed normal liver architecture with no evidence of fibrosis (on reticulin stain) but with intrahepatic cholestasis. Biliary ductules visualized in the portal area (Figure 1) showed multiple rosette formations without any interface activity, leading to a diagnostic dilemma. Thus, based on the above, viral, autoimmune, drug-induced liver injury (DILI) and Wilson’s etiology were ruled out. Therefore, we considered the first episode of benign cholestatic non-fibrotic disease as a differential. To confirm this, Whole Exome Sequencing (WES) was done. WES detected a nonsense/missense mutation at Exon 5 and another pathologic mutation leading to a stop codon and truncated protein at Exon 4 of the TJP2 gene. These findings were consistent with a diagnosis of PFIC-4.

**Table 1:** Showing interpretation of Next Generation Sequencing of the patient showing a mutation in Exon 5 and 4, diagnosis of disease as PFIC-4 involving TJP-2 protein.*The classification of the variants is done based on American College of Medical Genetics.

Gene	Location	Variant	Zygosity	Disease	Inheritance	Classification
TJP2 +	Exon 5	(c.847C>T)	Likely compound heterozygous*[5]	Progressive familial intrahepatic cholestasis-4	Autosomal recessive	Pathogenic
	Exon 4	(c.310G>T)				Uncertain significance

Following this, the patient was started on 300 mg of ursodeoxycholic acid (UDCA) twice daily. The patient’s symptoms improved gradually over six weeks, with a significant biochemical response. On follow-up after six months, the patient is asymptomatic and advised to continue Tab UDCA (Table 2).

**Table 2:** The blood investigation chart of the patient from admission shows improvement in liver function test after starting ursodeoxycholic acid (UDCA) and complete normalization of liver function test after six months of follow-up. WBC: White blood cell, SGOT: Serum glutamic-oxaloacetic acid transaminase, SGPT: Serum glutamic pyruvic transaminase, ALP: Alkaline phosphatase, PT/INR: Prothrombin time/international normalized ratio

Investigations	At Presentation	Day 3	Day 1 of UDCA therapy	Day 7 of UDCA therapy	6 months of UDCA Therapy
Hemoglobin (12-16 g/dl)	9.6	9.9	9.6	10.1	12
Platelets (150-500 x10^9^/L)	388k	298k	310k	290k	220k
WBC (4-12 k/ micro L)	8.4k	6.4k	7k	8k	6.4k
T Bilirubin (1-1.2 mg/dl)	16.6	18.4	20.4	12.2	0.4
D. Bilirubin (0-0.3 mg/dl)	12	14.3	14.8	8.9	0.1
SGOT (0-40 IU/L)	46	62	62	52	35
SGPT (0-40 IU/L)	27	34	37	34	49
ALP (50-120 IU/L)	292	369	379	290	140
Protein (6.5-7.5 g/dL)	6	6.2	6.4	6.5	7
Albumin (3.4-5.4 g/dL)	3.3	3.5	3.7	3.8	4.2
PT/INR	12.6/0.90	11/0.89	10/0.9	10/0.8	11/1.1
S. Creatinine (0.5-1 mg/dl)	0.9	0.8	0.98	0.7	0.8

## Discussion

In chronic intrahepatic cholestatic disorder, initial injury to the biliary epithelium causes poor bile acid transport from the liver to the gut, affecting any age. A thorough medical history is essential. These illnesses frequently result from hereditary or congenital problems in children. Familial intrahepatic cholestasis, including Benign Recurrent Intrahepatic Cholestasis (BRIC) and Progressive Familial Intrahepatic Cholestasis (PFIC), have been discovered as a result of significant advancements in the understanding of mechanisms of bile secretion at the cellular and molecular level. The cholestasis that characterizes these uncommon autosomal recessive illnesses is due to the mutations in the hepatic transport system genes involved in bile production. However, the precise incidence of PFIC is unknown, and it is expected to affect between one in 50,000 and one in 100,000 infants. Three categories have been recognized: presenting early in infancy are PFIC1 and PFIC2 while presenting later in infancy or adulthood is seen in PFIC3 [6].

GGT activity remains normal in PFIC1 and PFIC2 but high in PFIC3. Defects in the ATP8B1 gene affect bile salt production and cause PFIC1. ABCB11 gene defect, which impacts bile salt export, is the cause of PFIC2. Defects in the ABCB4 gene cause PFIC3, which impairs phospholipid production in the biliary system.

There have been descriptions of more recent PFIC variations in addition to the conventional kinds. Among them is PFIC4, which is associated with the altered expression of the tight junction protein (TJP2) gene. NR1H4 gene mutations cause PFIC5, while those in the MYO5B gene generate PFIC6 or MYO5B-related cholestasis.

**Figure 2:**
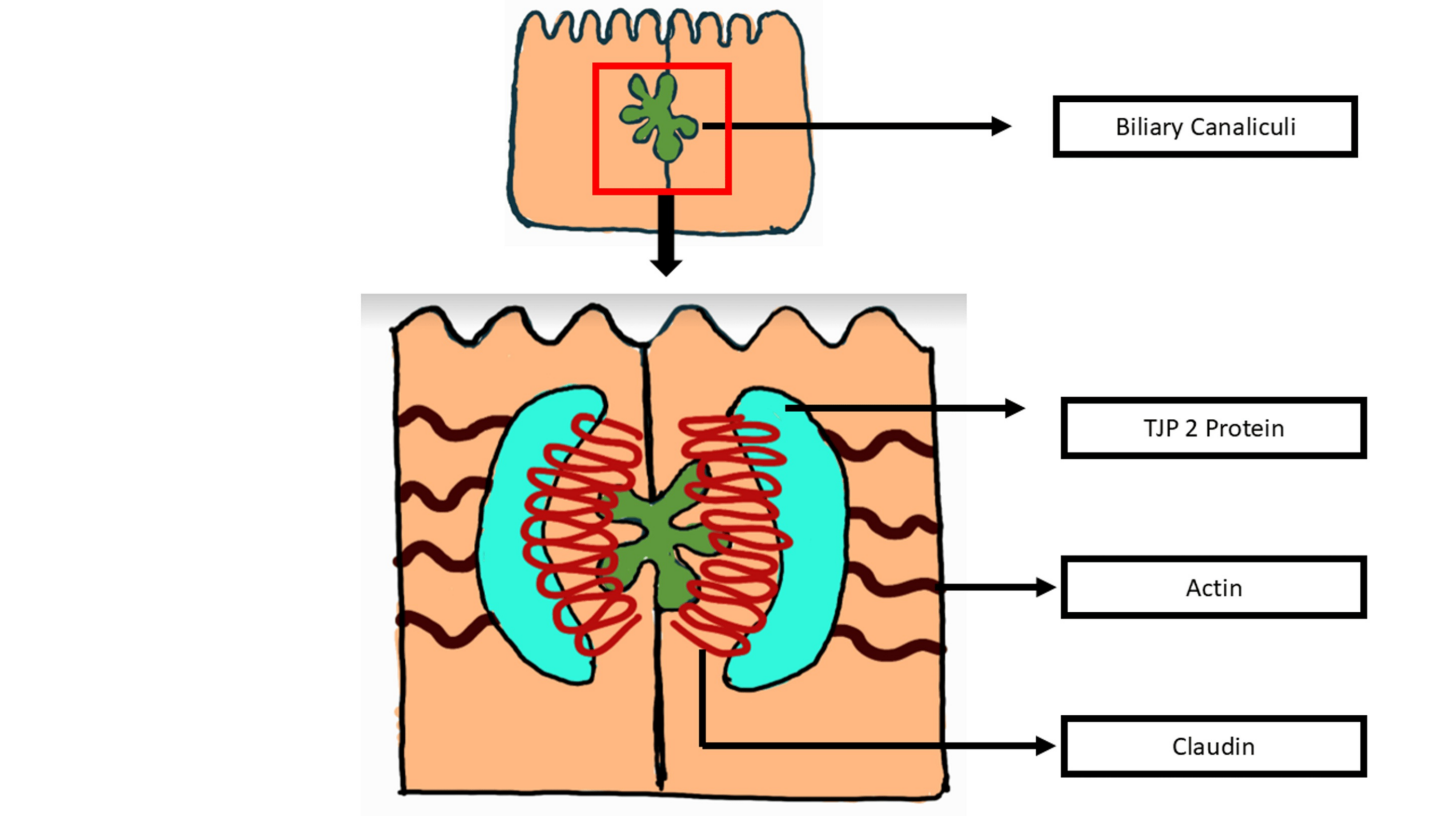
Structure of tight junctional protein 2 located on the long arm of chromosome 9 showing the relation of TJP2 protein with actin and claudin Figure made by author

The TJP2 gene on chromosome 9q21, first identified by Gumbiner et al. in 1991 [7], encodes for tight junction protein 2 (zona occludens-2), which links the transmembrane tight junction proteins such as claudin 1 and claudin 2 (CLDN1, 2), which are located on the bile canalicular membrane, to the actin cytoskeleton [8]. TJP2 mutations prevent CLDN1 from localizing to this membrane, reducing its integrity and allowing toxic bile acids to leak into hepatocytes and cause damage and cholestasis. Systemic symptoms secondary to central nervous and respiratory system involvement may be seen with the TJP2 mutation [9]. Liver damage is exacerbated by the detergent action of bile, leading to primary hepatic symptoms. TJP2 function is down-regulated in various tumor types, which could suggest a direct involvement in carcinogenesis [4]. A case of HCC connected to Tight-Junction Protein 2 Deficiency was described by Shengmei Zhou et al. [10] (Figure 2).

The condition presents in various ways, including mild non-jaundiced illness, recurring jaundice, and severe progressive liver disease [9-11] in some Amish patients with familial hypercholanemia, a single incompletely penetrant homozygous missense mutation that affects both isoforms of TJP2. This mutation results in pruritus and fat malabsorption, but not progressive liver disease, due to impaired TJP2 binding to claudins [12]. In addition, less severe TJP2 mutations have been reported in intrahepatic cholestasis of pregnancy [13].

Previous case reports and genetic data

The most extensive series on PFIC 4 is documented by Sambrotta et al. [11] and Zhang et al. [14]. In the case series documented by Sambrotta et al. [11], which included 12 patients, therapy in the form of a liver transplant was required amongst 9 cases (75%), with 2 (16%) cases developing portal hypertension. In contrast to Zhang et al. [14], who reported 7 cases, none required LT, and most responded well to conservative medical treatment for cholestasis. Zhang et al. also observed that severe presentations resulted from “truncating or canonical splice-site bi-allelic TJP2 mutations” [14], leading to a complete loss of protein expression. In this study, growth failure was seen in children with severe mutation, whereas sustained relief from pruritus with medical management and normal growth was exhibited amongst those with missense mutation. Complete loss of function is expected in those with a complete halt of protein translation secondary to homozygous mutation [11]. Milder severity of clinical manifestation is seen amongst those with missense and frame deletion [14]. A phenotype and genotypic correlation exist depending on the residual TJP2 activity.

Individuals with PFIC-4 are at risk of developing primary liver malignancy, as seen in PFIC-2. They may either present as liver masses or be diagnosed on histological examination of the liver explant. There is a critical need for vigilant monitoring and consistent follow-up care [15-16].

The PFIC registry in India examined the correlation between genotype and clinical course and prognosis amongst children with TJP2 deficiency-related cholestasis [17]. Of 278 patients with intrahepatic cholestasis secondary to genetic cause, 44 cases (15.8%) were identified to have TJP2 mutation. PFIC 4 patients can be classified based on their genetic mutations into three genetic types: TJP2-A: Both the alleles show missense mutation; Type B: One allele shows missense mutation while the other shows protein truncation mutation (PTM); TJP2-C: Both the alleles show PTM.

A case report from Nida et al. [18] reported a 15-year-old male with PFIC4 with a novel compound heterozygous missense mutation in exon 17 of the TJP2 gene. Another case report from Ting Ge et al. [19] reported a novel compound heterozygous mutation c.2448+ 1G > C/c.2639delC (p. T880Sfs*12) in TJP2 by Next Generation Sequencing.

**Figure 3:**
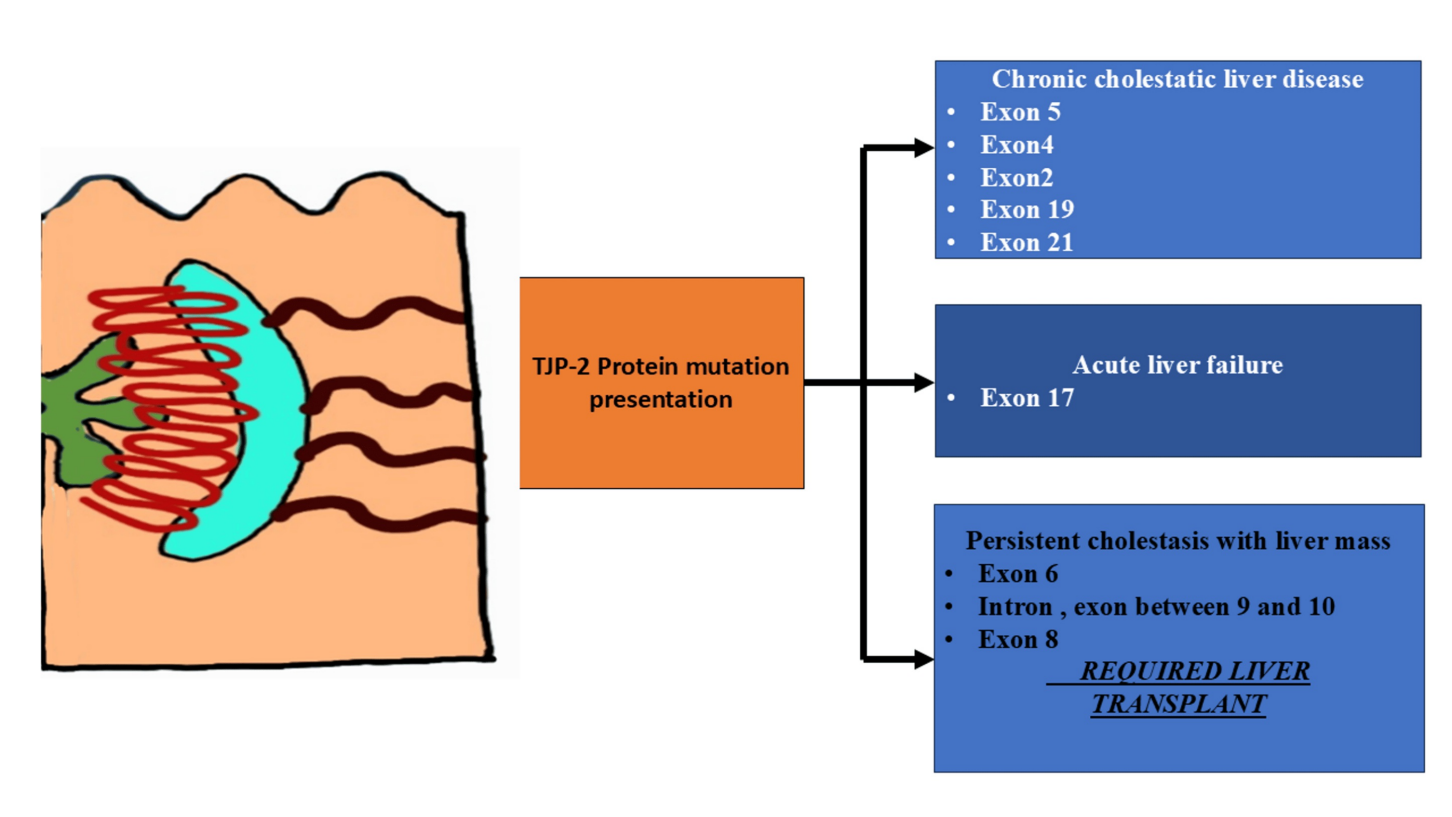
Showing different exon and intron mutations associated with PFIC-4 involving TJP-2 protein (Figure made by author)

Our case had no evidence of growth retardation, portal hypertension, or evidence of chronic liver disease on liver biopsy or VCTE; paradoxically, her jaundice resolved and LFT normalized, which can be explained by her heterozygous mutation of the TJP2 gene. However, a case report by Nida et al. [18] reported a heterozygous mutation of the TJP2 protein presenting as portal hypertension and chronic liver disease. The family of our patient has been counseled about the genetic nature of the disease and informed about the need to keep the patient under surveillance, as there is a risk of developing liver malignancy in the future (Table 3, Figure 3).

**Table 3:** Showing different case reports of PFIC-4 with genetic mutation and clinical presentation UDCA: Ursodeoxycholic acid

Study	Intron /Exon	Homozygous /Heterozygous	Sequence	Age at diagnosis	Chronic liver disease	Presentation	Management and response to treatment
Our study	5 Exon 4 Exon	Compound Heterozygous	(c. 847C>T) (c.310G>T)	13 years	No	Cholestatic liver disease	Conservative treatment with T UDCA and nutritional support.
Shengmei Zhou et al. [10]	Intron between exon 9 and 10 8 Exon	Compound Heterozygous	c. 2668-1G>T /c.2438dupT	26 months	LT	Persistent cholestasis with acute liver failure with liver mass	Failure of conservative treatment required a liver transplant (LT).
Shengmei Zhou et al. [10]	6 Exon	Homozygous	r c.817delG	24month	LT	Persistent cholestasis with Liver mass	Failure of conservative treatment required LT.
Nida et al. [18]	17 Exon	Compound Heterozygous	g.71854869T>C	15 years	Yes	Cholestatic liver disease	Conservative treatment with T. UDCA, beta blocker and nutritional support.
Ge T, Zhang X, et al. [19]	19 Exon 21 Exon	Heterozygous	c.2448 + 1G > C c.2639delC	23 months	No	Persistent jaundice	Conservative treatment with T UDCA and nutritional support.
Hana Halibi et al. [20]	17 Exon	Homozygous	(C.2524C >T)	46 days	LT	Acute liver failure	Failure of conservative treatment required LT.
H. Meshram et al. [21]	2 Exon	Homozygous	(c.157 c >4)	3.5 years	No	Cholestatic liver disease	Conservative treatment with nutritional support.

## Conclusions

PFIC is a challenge for clinicians due to its uncommon occurrence. When adolescents present with unexplained cholestasis, PFIC 4 should be kept on the list of differential diagnoses. Early identification, symptomatic management, nutritional support, and regular HCC screening are advised. A correlation exists between the genotype and phenotype, determined by the residual functional TJP2 activity level.
